# Synergistic cytotoxicity of olive leaf extract-loaded lipid nanocarriers combined with Newcastle disease virus against cervical cancer cells

**DOI:** 10.1371/journal.pone.0308599

**Published:** 2024-08-14

**Authors:** Arash Golalipour, Ali Mohammadi, Saeid Hosseinzadeh, Alireza Soltani, Vahid Erfani-Moghadam

**Affiliations:** 1 Department of Pathobiology, School of Veterinary Medicine, Shiraz University, Shiraz, Iran; 2 Department of Food Hygiene and Public Health, School of Veterinary Medicine, Shiraz University, Shiraz, Iran; 3 Golestan Rheumatology Research Center, Golestan University of Medical Science, Gorgan, Iran; 4 Medical Cellular and Molecular Research Center, Golestan University of Medical Sciences, Gorgan, Iran; 5 Department of Medical Biotechnology, School of Advanced Technologies in Medicine, Golestan University of Medical Sciences, Gorgan, Iran; Icahn School of Medicine at Mount Sinai Department of Pharmacological Sciences, UNITED STATES OF AMERICA

## Abstract

Despite recent medical progress, cervical cancer remains a major global health concern for women. Current standard treatments have limitations such as non-specific toxicity that necessitate development of safer and more effective therapeutic strategies. This research evaluated the combinatorial effects of olive leaf extract (OLE), rich in anti-cancer polyphenols, and the oncolytic Newcastle disease virus (NDV) against human cervical cancer cells. OLE was efficiently encapsulated (>94% loading) within MF59 lipid nanoparticles and nanostructured lipid carriers (NLCs; contains Precirol as NLC-P, contains Lecithin as NLC-L) to enhance stability, bioavailability, and targeted delivery. Physicochemical analysis confirmed successful encapsulation of OLE within nanoparticles smaller than 150 nm. In vitro cytotoxicity assays demonstrated significantly higher toxicity of the OLE-loaded nanoparticle formulations on HeLa cancer cells versus HDF normal cells (P<0.05). MF59 achieved the highest encapsulation efficiency, while NLC-P had the best drug release profile. NDV selectively infected and killed HeLa cells versus HDF cells. Notably, combining NDV with OLE-loaded nanoparticles led to significantly enhanced synergistic cytotoxicity against cancer cells (P<0.05), with NLC-P _(OLE)_ and NDV producing the strongest effects. Apoptosis and cell cycle analyses confirmed the increased anti-cancer activity of the combinatorial treatment, which induced cell cycle arrest. This study provides evidence that co-delivery of OLE-loaded lipid nanoparticles and NDV potentiates anti-cancer activity against cervical cancer cells in vitro through a synergistic mechanism, warranting further development as a promising alternative cervical cancer therapy.

## 1. Introduction

Cervical cancer ranks as the fourth most common and deadly cancer affecting women worldwide [1,2]. The primary cause of this disease is infection with the human papillomavirus (HPV), predominantly types 16 and 18 [3,4]. Increased expression of the virus’s E6 and E7 oncoproteins, through various molecular mechanisms that interfering with host cell functions, leads to inhibited apoptosis and ultimately tumor formation [5]. Despite advancing medical technologies, cervical cancer remains a leading cause of cancer-related deaths among women [2]. Current treatment modalities, including surgery, chemotherapy, radiotherapy, and immunotherapy face limitations due to high costs, lack of specificity, and numerous side effects. Consequently, there is a pressing need to develop novel, safe therapeutic approaches with improved efficacy. In recent years, the induction of apoptosis has emerged as a crucial therapeutic target and a promising strategy to inhibit the proliferation of cancer cells [6]. This approach offers potential for developing more effective and targeted treatments for cervical cancer, addressing the limitations of current therapies and potentially improving patient outcomes.

In this context, herbal phytochemicals have shown significant potential in cancer prevention and treatment. In particular, plant polyphenols have demonstrated anti-carcinogenic effects, primarily through their antioxidant activity, modulating of oxidative stress, and ability to alter cell signaling and gene expression related to inhibiting cell proliferation and promoting apoptosis [7,8]. These compounds are considered promising therapeutic aids due to their high cytotoxicity towards cancer cells, biocompatibility, minimal side effects, and low cost. Olive leaves are rich in phenolic compounds, including oleuropein, tyrosol and hydroxytyrosol, which possess antioxidant, antimicrobial, anti-inflammatory, and anti-carcinogenic properties [9,10]. The Phenols present in olive leaf extract (OLE) have been shown to inhibit cell proliferation and induce apoptosis in various cancer cell lines, making them a subject of interest in cancer research and potential therapeutic development [11,12]. Despite their potential, OLE polyphenols suffer from poor stability in environmental and biological conditions. To address this issue, innovative strategies are required to enhance their stability, bioactivity, and bioavailability, while also protecting them against systemic degradation or modification and improving their delivery to cancer cells. One promising approach to overcome these limitations and boost the efficiency and safe delivery of OLE to target cells is encapsulation within biodegradable nanocarriers. This technique could potentially preserve the beneficial properties of OLE polyphenols while addressing the challenges associated with their direct use in therapeutic applications [13,14]. MF59 is an oil-in-water nanoemulsion adjuvant developed by Novartis that consists of squalene oil, Span 85, and Tween 80 emulsified in a citrate buffer. Due to its small particle size of around 100–150 nm, MF59 has been shown to effectively enhance the immune response to vaccine antigens by facilitating antigen trafficking to lymph nodes. MF59 has also attracted interest as a delivery system for therapeutic compounds due to its ability to efficiently encapsulate both hydrophilic and hydrophobic drugs [15]. Also, Nanostructured lipid carriers (NLCs) are a type of nanoparticle composed of a solid heterogenous lipid matrix. In NLCs, both solid and liquid lipid phases co-exist simultaneously, which increases the drug loading capacity and results in improved bioavailability. They can effectively deliver both hydrophobic and hydrophilic drugs in a controlled manner for extended periods of time, making them a promising drug delivery system [16,17]. Both MF59 and NLCs are promising nanocarrier systems, but their relative encapsulation and release properties for olive leaf extract needed to be characterized. One of the objectives of this study is to evaluate the potential of MF59 and NLCs to deliver olive leaf extract polyphenols to cervical cancer cells.

On the other hand, oncolytic virus therapy, a form of virotherapy, is emerging as a promising approach for treating various human diseases, including cancer. Oncolytic viruses are natural or genetically engineered viral strains that selectively proliferate in cancer cells and spread without harming normal cells, leading to induction of oncolysis in them with the aim of stabilizing and reducing tumor progression. This selectivity is attributed to defects in the host’s immune system, particularly the deficiency of the interferons signaling pathway in cancer cells [18]. This characteristic allows oncolytic viruses to exploit vulnerabilities specific to cancer cells. Numerous oncolytic viruses have been investigated for cancer therapy [19]. Among these, the Newcastle disease virus (NDV) has shown significant potential. NDV is a naturally oncolytic virus belonging to Paramyxoviridae family. It causes acute disease in avian species but is non-pathogenic in mammals, including humans. NDV has demonstrated a high capacity for destroying cancer cells [20]. The mechanism of action for NDV begins with its adherence to malignant cells, followed by entry through receptor-mediated endocytosis. Studies have revealed that NDV exhibits multifactorial anticancer effects, including: stimulating the host immune response to identify and attack tumors, induction of apoptotic pathways in cancer cells via viral proteins such as hemagglutinin-neuraminidase (HN) glycoproteins, and direct lysis of cancer cells through viral replication [21]. Several studies have investigated the use of NDV as a potential cancer treatment, reporting efficacy without significant side effects in preclinical and early clinical trials [22,23]. These findings underscore NDV’s potential as a promising candidate for further development in cancer therapy, offering a novel approach to targeting and eliminating cancer cells while minimizing harm to healthy tissue.

Given the complex nature of cancer, researchers have been focusing on combination therapies as potential treatment strategies. One promising approach involves combining biochemical compounds derived from natural sources, such as plants, with virus-based methods. While the effects of OLE and NDV on cervical cancer cells have been studied separately, their combined impact remains unexplored. This study aimed to investigate the therapeutic effect of NDV (LaSota strain) in combination with OLE-loaded lipid nanoparticles on HeLa cervical cancer cells in vitro. We hypothesized that co-delivering NDV with OLE-loaded nanoparticles would result in a synergistic response against cancer cells. By evaluating this novel approach using HeLa cancer cells in vitro, our research seeks to explore a potentially safer and more effective alternative for the management and treatment of cervical cancer. This study serves as a foundation for understanding the potential benefits of combining these two distinct therapeutic agents in the fight against cervical cancer.

## 2. Materials and methods

### Chemicals

Span 85 and Tween 80 (as non-ionic surfactants), and Squalene were obtained from Sigma-Aldrich Co (USA). Lecithin was procured from Merck Co (Germany). Precirol ATO 5 (glyceryl distearate) was obtained from Gattefossé (France). Olive leaf extract (*Olea europaea L*.) with an oleuropein concentration of 6.75 mg/mL (equivalent to 12500 μM) was purchased from Dr. Soleimani’s Giah Essence Pharmaceutical Company (Golestan, Iran). Ultrapure water (Millipore, USA) was used in all necessary steps.

### Cell culture reagents

Human cervical cancer (HeLa) and human dermal fibroblast (HDF) cell lines were obtained from the Pasteur Institute, Iran. Dulbecco’s Modified Eagle’s Medium (DMEM), fetal bovine serum (FBS), penicillin-streptomycin and trypsin-EDTA were purchased from Gipco (UK). Dimethyl sulfoxide (DMSO) and 3-(4,5-dimethylthiazol-2-yl)-2,5 diphenyl-tetrazolium bromide (MTT) reagent was provided by Sigma- Aldrich (USA). Annexin V-FITC and Propidium iodide (PI) were procured from Biolegend (USA).

### Preparation of nanoparticles

#### Preparation of MF59

Briefly, the preparation of the MF59-based lipid nanocarrier involved two phases: a lipid phase and an aqueous phase. The lipid phase contained 4.3% (w/v) squalene and 0.5% (w/v) Span 85. This was added dropwise over 30 min to the aqueous phase using a high-speed homogenizer (Ultra-Turrax T25, IKA, Germany) operating at 12,000 rpm. The aqueous phase contained 0.5% (w/v) Tween 80, 1 mL of citrate buffer (containing citric acid monohydrate and trisodium citrate dihydrate, pH 6.5), and pure water to a final volume of 10 mL. Following homogenization, the mixture was sonicated for 30 s at 20 W using a sonicator (XL-2000, QSonica Sonicator, USA) to ensure proper dispersion.

#### OLE-Loaded MF59

To load OLE in MF59, 1.5 mL of filtered OLE (equivalent to 18.75 mM oleuropein) was added directly to the aqueous phase before the addition of the lipid phase and homogenization. The final concentration of OLE in the 10 mL total volume of the manufactured nanocarrier was 1.875 mM.

#### Preparation of NLCs

NLCs were prepared using a modified MF59 composition and hot melt homogenization method. The lipid phase contained of precirol (or lecithin in an alternative formulation) and squalene in three different ratios (70:30, 60:40, 50:50), totaling 4.3% (w/v). The aqueous phase comprised 0.5% (w/v) Span 85, 0.5% (w/v) tween 80, 1 mL of citrate buffer, and purified water to a final volume of 10 mL. precirol, as solid lipid, was heated in an incubator to 5°C above its melting point (56°C) to achieve a clear and homogeneous phase (The same process was applied to lecithin) [24,25]. To prevent rapid solidification, the lipid phase was maintained at 55°C. The aqueous phase was then slowly added to the molten lipid phase, and the mixture was immediately sonicated using a sonicator (XL-2000, QSonica Sonicator, USA) for 4 cycles (30 s on, 15 s off) at 20 W. The resulting nanoemulsion was kept under magnetic stirring at room temperature for 15 min, then immediately stored at 4°C for 3 h to allow lipid recrystallization and NLCs formation. The final concentration of OLE in the 10 mL is equal to 1.875 mM.

#### OLE-Loaded NLCs

To load OLE in NLCs, 1.5 mL of filtered OLE was placed in an incubator at 55°C. To preserve the integrity and stability of OLE, it was mixed into the aqueous phase just prior to adding the molten lipid phase and the sonication process. This new phase was then added to the molten lipid phase, and the mixtures were immediately sonicated for 4 cycles of 30 s on and 15 s off at 20 W (XL-2000, QSonica Sonicator, USA).

### Physicochemical characterization of nanoparticles

#### Size and Zeta potential

The prepared nanoparticles were diluted up to 50 times with ultrapure water to prevent multiple scattering effects; the mean particle size, polydispersity index (PDI), and zeta potential of nanoparticles were measured by dynamic light scattering (DLS) using a particle size analyzer (Microtrac^®^ MRB, Nanotrac Wave II, Canada). Each sample was measured in triplicate. The data were reported as the mean ± standard deviation (SD) and the average particle size based on volumetric diameter.

#### UV-Visible spectroscopy

The optical absorption spectra of the physical mixture of nanocarriers were investigated. The absorbance of empty nanocarriers and nanocarriers containing OLE was measured using a UV-visible spectrophotometer (DeNovix DS-11, USA) over a wavelength range of 200–800 nm.

#### Chemical structure analysis

Fourier transform infrared (FT-IR) spectroscopy is a precise technique for identifying functional groups, elucidating the structure of organic compounds, and providing insights into the nature of interactions between nanoparticle components. In this study, employing direct sampling, FT-IR spectra were acquired using an IR spectrometer (Spectrum RX1) over a wavenumber range of 400–4000 cm^−1^. For spectroscopic analysis, pellets were prepared by combining 300 mg of potassium bromide with 2 mg of each sample, including OLE and its carrier nanoparticles (which were lyophilized for 72 h).

#### Morphology analysis

The surface morphology of the nanoparticles was characterized using a scanning electron microscope (SEM; Zeiss EVO 50, Germany) operated at an accelerating voltage of 10 kV. For this analysis, diluted samples were deposited onto brass stubs and desiccated in an incubator at 40°C. Subsequently, the samples were sputter-coated with a thin layer of gold to enhance conductivity prior to being mounted on the SEM sample holder and subjected to electron beam scanning.

#### Encapsulation efficiency

The encapsulation efficiency (EE) of OLE-loaded nanoparticles was quantified by measuring the concentration of free OLE in the dispersion medium, based on oleuropein content. Oleuropein, the predominant and most bioactive polyphenol in OLE (constituting approximately 85%), can be identified and quantified using UV spectroscopy. Thus, the oleuropein spectrum serves as a suitable proxy for determining OLE concentration. In this study, encapsulation efficiency was assessed using two methods: centrifugation and dialysis. A standard calibration curve was generated by measuring the absorbance of various OLE dilutions at 234 nm (the maximum absorbance wavelength of oleuropein) using a UV-visible spectrophotometry (DeNovix DS-11, USA). The encapsulation efficiency was calculated based on the initial amount of oleuropein incorporated into each formulation, using the following equation:

$$EE\left(\%\right)=\frac{Amount\;of\;initial\;added\;OLE-Amount\;of\;free\;OLE}{Amount\;of\;initial\;added\;OLE}\times 100$$

#### In vitro drug release

A comprehensive quantitative and qualitative analysis of OLE release kinetics from MF59 and NLCs was conducted using a dialysis membrane method. The experiments were performed at pH 5.4 and 7.4 at room temperature, simulating the microenvironments of cancer and normal cells, respectively. The release of oleuropein from OLE-containing nanoparticles was evaluated in phosphate buffered saline (PBS) at predetermined time intervals (12, 24, 48, 86, and 132 h post nanoparticle preparation) using a cellulose dialysis membrane with a molecular weight cut-off (MWCO) of 12,000–14,000 Da. Quantification of released oleuropein was performed using UV-visible spectrophotometer at 234 nm. The results are expressed as the percentage of OLE released relative to the total OLE initially encapsulated in the nanoparticles. All measurements were conducted in triplicate for each sample to ensure reproducibility.

#### Cell culture

Hela (cervical cancer cell line) and HDF (normal human dermal fibroblast cell line) cells were cultured in DMEM supplemented with 10% FBS, 100 U/mL penicillin-streptomycin, and incubated at 37°C in a humidified atmosphere containing 5% CO2. The cells used in this study were between passages 5–15. The cells were allowed to reach approximately 90% confluence before trypsinization using 0.25% Trypsin-EDTA. Subsequently, cells were seeded in plates for individual experiments.

#### NDV infectious dose assay

The oncolytic virus used in this study was an attenuated LaSota strain of NDV. A commercial live virus vaccine of the LaSota strain was purchased from Dechra Pharmaceutical Group (GENERA, Croatia). The infectious dose of this virus was determined using the 50% tissue culture infectious dose (TCID50) assay. Briefly, HeLa and HDF cell lines were cultured in 96-well plates (10^4^ cells/well) in a final volume of 100 μL of DMEM supplemented with 10% FBS and 1% penicillin-streptomycin. Cells were incubated for 24 h at 37°C in a 5% CO_2_ atmosphere. Subsequently, the cells were infected with serial dilutions (10^−1^ to 10^−8^) of the LaSota strain of NDV prepared in culture medium. Seven wells were used for each viral dilution, and they were incubated under the same conditions as before. The cells were then evaluated daily for 5 days using an inverted microscope to observe cytopathic effect (CPE). After recording the results, viral titers of propagated viruses were calculated using the TCID50 method based on the Spearman–Kärber algorithm (Hierholzer and Killington, 1996).

#### Cytotoxicity assay

The cytotoxicity of empty nanoparticles (MF59, NLC-P, NLC-L), nanoparticles loaded with OLE (MF59 _(OLE)_, NLC-P _(OLE)_, NLC-L _(OLE)_), the LaSota strain of NDV, and their combination effects were evaluated using the MTT cell proliferation assay in HeLa and HDF cell lines. The cells were seeded in 96-well plates at a density of 10,000 cells/well and incubated at 37°C under 5% CO_2_ with saturating humidity for 24 h. The cells were then treated with free OLE and OLE-loaded nanocarriers at concentrations of 18.75, 37.5, 75, 150, 300, 450 and 600 μM (Based on the oleuropein concentration in OLE) and empty nanocarriers (equivalent values) under the mentioned conditions for 24 and 48 h [26]. Treatment with DMSO was considered as the negative control and medium without treatment as the positive control. A weak and lentogenic LaSota strain of NDV was used for virotherapy in this study. To assess NDV cytotoxicity, the cell lines were infected with NDV at multiplicities of infection (MOI) of 0.1, 0.2, 0.4, 0.8, 1.2, and 1.6 for 48 h under the previous conditions. To investigate the cytotoxicity of the combination treatment of NDV and OLE-loaded nanoparticles, the cell lines were infected with NDV (MOI 0.4 and 0.8) along with OLE-loaded nanoparticles at different concentrations (18.75, 37.5, 75, 150, 300 and 450 μM) for 48 h under the above conditions. After treatment, cells were washed with PBS and incubated with 100 μL of 1 mg/mL MTT solution for 3 h under light-protected conditions. The MTT solution was then removed and 100 μL of DMSO was added to each well to dissolve the purple formazan crystals produced by the mitochondrial dehydrogenase enzymes of living cells. To assess cytotoxic effects of the treatments, optical absorbance was measured at 570 nm with a reference wavelength of 630 nm using a microplate reader (Stat Fax 4300-Chromate, USA). Untreated cells were considered as 100% viability and the percentage of relative cell viability was calculated as (A570 of treated sample / A570 of untreated sample) × 100. The IC50 values were also calculated relative to untreated control cells. All treatments were evaluated in triplicate in both cell lines.

#### Apoptosis assay

Apoptosis induction in HeLa cells undergoing various treatment was assessed using an Annexin V-FITC/PI staining technique followed by flow cytometry analysis, according to manufacturer’s instructions (Biolegend, CA, USA). Briefly, HeLa cells were seeded in 6-well plates at a density of 50,000 cells/well and incubated at 37°C under 5% CO_2_ with saturated humidity for 24 h. Then the cells were infected with NDV at an MOI of 0.4, as well as treated with free OLE and its carrier nanoparticles at a concentration of 100 μM (as the minimum determined IC50 value) separately and in combination with each other under the previous conditions for 48 h. After treatment, the cells were harvested, washed twice with cold PBS, and resuspended in 100 μL of Annexin V binding buffer (Biolegend, CA, USA). Next, 5 μL of Annexin V-FITC and 10 μL of PI were added to the cell suspensions, and were incubated for 15 min at room temperature under light-protected conditions, finally, were diluted with 400 μL of binding buffer. The cells were analyzed using a flow cytometer (BD FACSCalibur, USA). Untreated cells served as controls. Data analysis was performed using FlowJo software (v10.0.7). All experiments were independently performed in triplicate.

#### Cell cycle assay

Flow cytometry was used to examine the distribution of cell cycle phases in HeLa cells that underwent various treatment conditions. HeLa cells were cultured and treated under the same conditions as the previous step. The cells were then harvested, washed twice with PBS and fixed with chilled 70% ethanol at 4°C for 3 h. The fixed cells were centrifuged at 500×g for 5 min at 4°C and washed twice with cold PBS. The cells were finally re-suspended in 500 μL PBS containing 50 μg/mL PI, 100 μg/mL RNase (DNAbiotech), and 4 μL Triton X100, and incubated for 30 min at room temperature under light-protected conditions. Cell fluorescence of the prepared samples was analyzed using a flow cytometer (BD FACSCalibur, USA). Data and histograms were evaluated using FlowJo software (v10.0.7). All experiments were independently performed in triplicate.

#### Ethics approval

The research has received approval from the ethics committee at Shiraz University and the Cellular and Molecular Medical Research Center at Golestan University of Medical Sciences (IR.GOUMS.REC.1401.075).

#### Statistical analysis

Statistical differences between experimental groups were analyzed using GraphPad Prism software (v9.5.1) via one-way or two-way analysis of variance (ANOVA), as appropriate. The results were considered statistically significant at p < 0.05. Data are presented as the mean ± SD.

## 3. Results

### Physicochemical analysis of nanoparticles

#### Size and Zeta potential

The physicochemical properties, including size and zeta potential, are important factors that determine the stability and solubility of nanoparticles. In this study, the means particle size, PDI, and zeta potential of MF59, NLC-P, and NLC-L nanoparticles were measured using DLS. Table 1 presents the average particle size, PDI, and zeta potential values obtained from DLS analysis for each type of nanoparticle formulation.

**Table 1:** Means of size, PDI, and zeta potential of MF59, NLC-P, and NLC-L nanoparticles.

Name	Size (nm)	SD	Width	PDI	Zeta potential (mv)
NLC-P_50_	114.7	± 61.7	123.5	0.614	- 3.2
NLC-P_60_	120.6	± 36.4	72.9	0.269	- 6.9
NLC-P_70_	154.2	± 60.5	121.1	0.1689	- 8.3
NLC-L_50_	92.1	± 26.7	53.5	0.301	- 2.4
NLC-L_60_	115.0	± 35.8	71.5	0.1249	- 5.9
NLC-L_70_	186.1	± 68.8	137.6	0.073	- 7.5
MF59	119.7	± 37.3	74.6	0.1222	- 6.7

NLC formulations were developed using varying ratios of precirol/lecithin to squalene in the lipid matrix: 70:30, 60:40, and 50:50%. Based on superior physicochemical properties including optimal particle size, zeta potential and PDI, the 60:40 ratio matrix with 60% precirol/lecithin (NLC-P_60_ and NLC-L_60_) and 40% squalene was selected for OLE encapsulation studies. In the following, for the convenience of presentation, we refrained from mentioning the number in NLC formulations. According to Table 1, the Physicochemical characterization showed all nanoparticle formulations had sizes between 100–150 nm, indicating appropriate characteristics. PDI values were less than 0.6, demonstrating homogeneous dispersion. Zeta potential was negative for all nanoparticles. The negative charge increased with greater percentage of precirol/lecithin in the lipid matrix. Table 2 presents the average physicochemical characterization data for the OLE-containing nanocarriers that were selected for subsequent investigation.

**Table 2:** Means of size, PDI, and zeta potential of nanocarriers loaded with OLE (MF59 _(OLE)_, NLC-P _(OLE),_ and NLC-L _(OLE)_).

Name	Size (nm)	SD	Width	PDI	Zeta potential (mv)
NLC-P _(OLE)_	121.8	± 33.7	67.4	0.392	- 12.4
NLC-L _(OLE)_	117.0	± 41.2	83.4	0.1158	-10.2
MF59 _(OLE)_	126.3	± 47.9	95.7	0.206	- 13.8

Based on the results, the obtained data were largely consistent with SEM micrographs. Upon addition of OLE, the size of the nanoparticles increased slightly. The PDI values were less than 0.4, indicating a highly homogeneous dispersion. The zeta potential of nanoparticles containing OLE showed an increased in negative charge, which may be attributed to the hydroxyl group present in OLE polyphenols.

#### UV spectrophotometry

**Fig 1:**
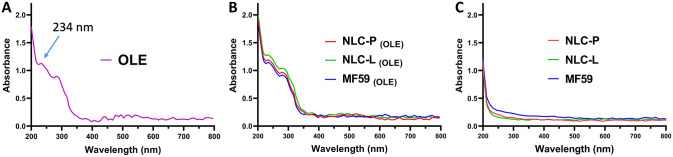
The UV-Vis absorption spectrum of free OLE (A), OLE-loaded nanoparticles (B), and empty nanoparticles (C).

To investigate the UV absorption spectrum of OLE-loaded nanoparticles, empty nanoparticles, and free OLE, dilutions with a concentration of 20 μM were prepared. The samples were evaluated using a UV-visible spectrophotometer (DeNovix, DS-11, USA) over the wavelength range of 200–800 nm. The results showed that in OLE and its carrier nanoparticles, the maximum optical absorption was obtained at 234 nm (λmax of oleuropein). The peak at 202 nm was observed for the pure NLC-P. The results of UV spectral analysis support the incorporation of OLE into the matrix of nanoparticles loaded with it (MF59 _(OLE)_, NLC-P _(OLE)_, NLC-L _(OLE)_). The spectra obtained from spectroscopic analysis of the samples are presented in Fig 1.

#### FT-IR analysis

**Fig 2:**
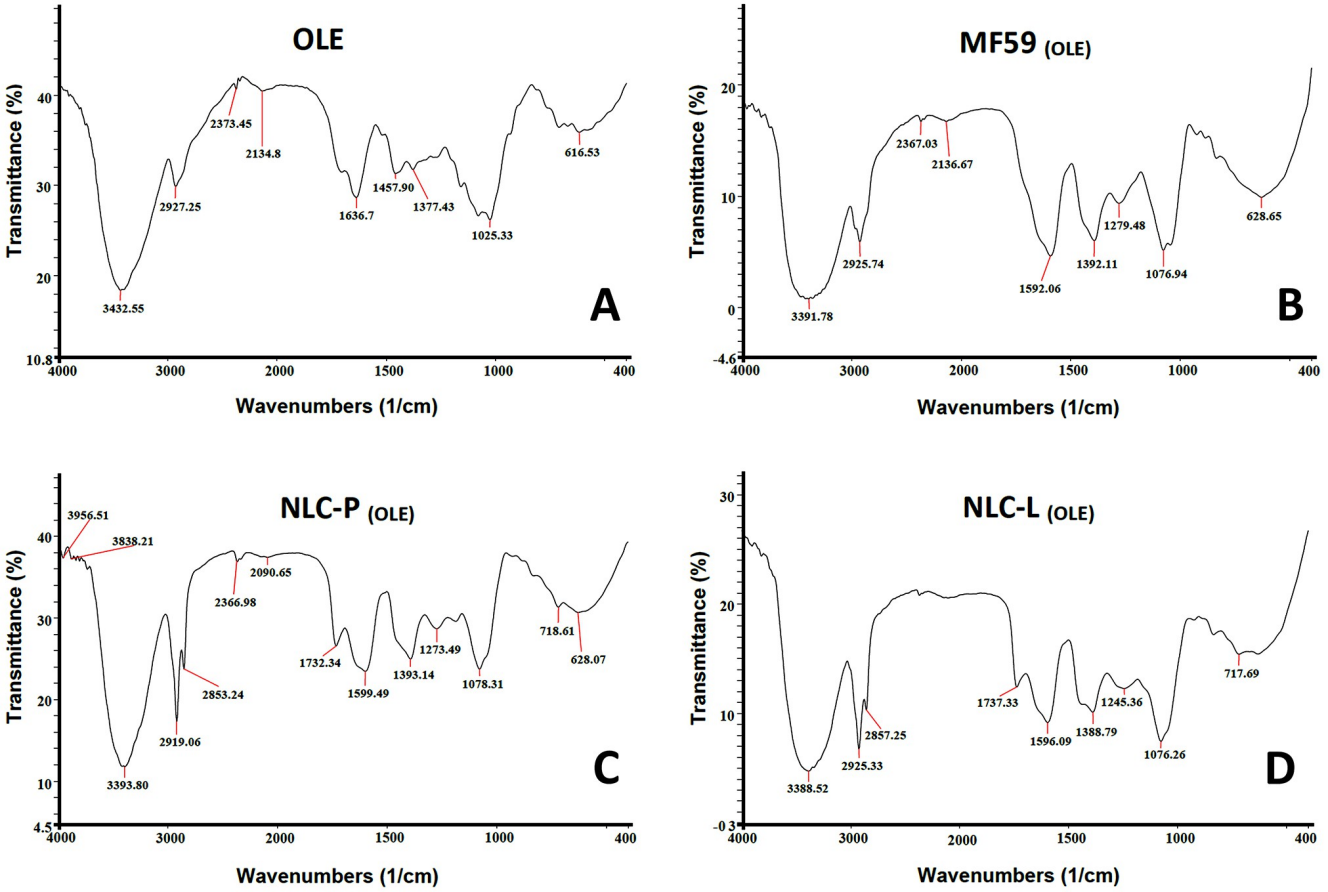
FT-IR spectra for free OLE (A) and nanoparticles loaded with OLE (B, C, D).

FT-IR spectroscopy is a highly effective technique employed to assess the chemical stability of different substances following their encapsulation in nanoparticles and also to determine the functional groups contained within them. Fig 2 depicts FT-IR spectra of free OLE and its carrier nanoparticles. As shown in the OLE spectrum, the carbonyl group (C = O) corresponding to the carboxylic acid or ester appeared at 1636 cm^-1^. A peak in the region of 2927 cm^-1^ corresponds to the C-H aliphatic group of OLE compounds. Also, the hydroxyl group (O-H) appeared at 3432 cm^-1^. In the Mf59 _(OLE)_ complex, peaks in the region of 1076 cm^-1^ and 1592 cm^-1^ correspond to the C-O ether group and the C-C group, respectively. Peaks in the region of 2925 cm^-1^ and 3391 cm^-1^ are related to the C-H group and hydroxyl group (O-H), respectively [24,27]. In the NLC-P _(OLE)_ complex, peaks in the region of 1078 cm^-1^ and 1599 cm^-1^ correspond to the C-O ether group and the C-C group, respectively. A peak in the region of 1732 cm^-1^ corresponds to the carbonyl group (C = O) and peaks in the region of 2853 cm^-1^ and 2919 cm^-1^ are associated with C-H. The hydroxyl group (O-H) appeared at 3393 cm^-1^. The NLC-L _(OLE)_ complex exhibits a peak around 1737 cm^-1^, indicating the presence of the carbonyl group (C = O). Nobari Azar et al. have shown the peaks at 1739 and 3393 cm^-1^ correspond to the hydroxyl and carbonyl groups of OLE encapsulated within NLC [28]. Peaks in the region of 1076 cm^-1^ and 1596 cm^1^ are associated with the C-O ether group and C-C group. Peaks in the region of 2857 cm^-1^ and 2925 cm^-1^ are associated with the C-H group. Also, the hydroxyl group (O-H) appeared at 3388 cm^-1^. The results of FT-IR show that peaks related to OLE polyphenols, especially the hydroxyl and carbonyl groups, can be seen in all nanocarriers.

#### Morphology analysis

**Fig 3:**
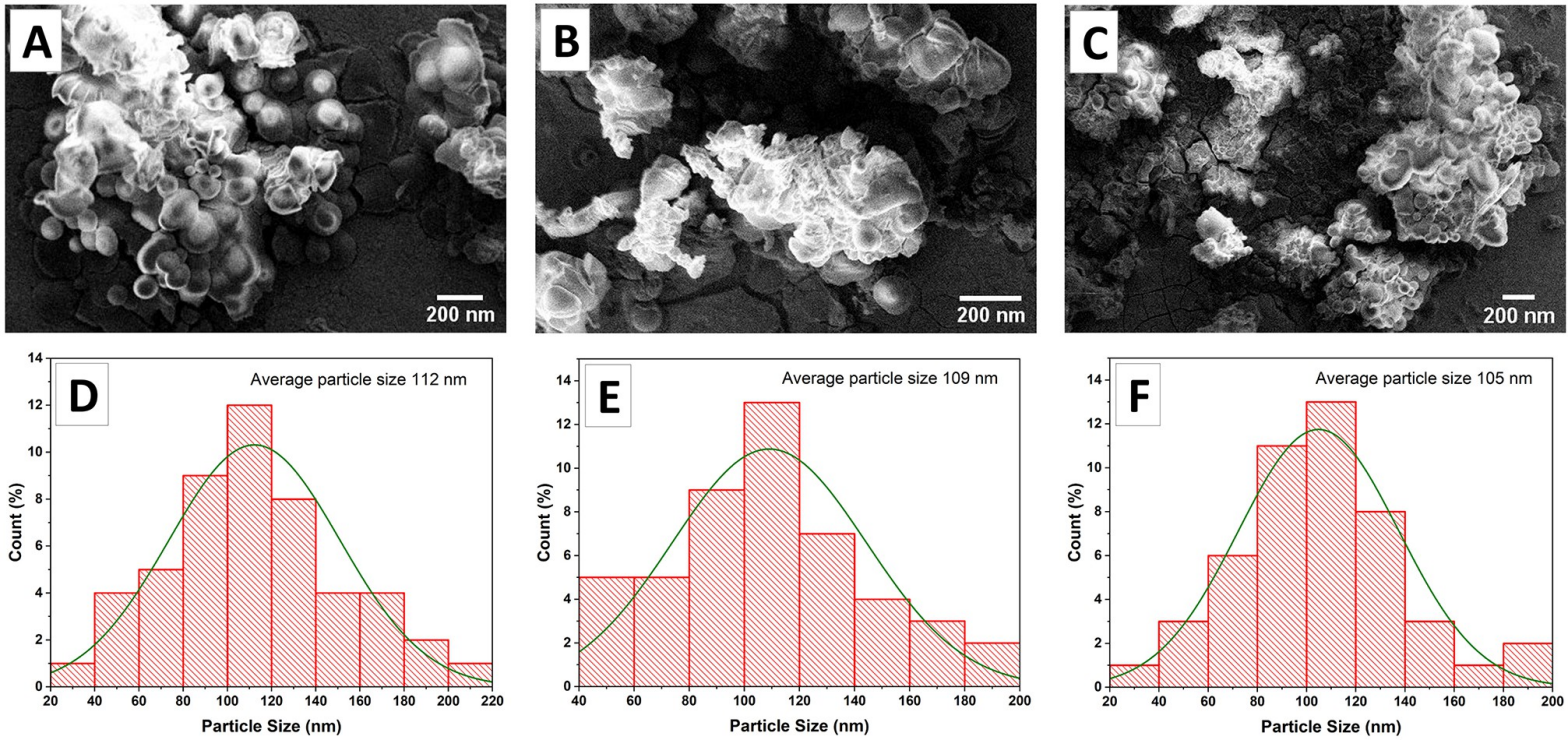
SEM image of MF59 (A), NLC-P (B) and NLC-L (C); Particle size distributions diagram of blank nanoparticles MF59 (D), NLC-P (E), NLC-L (F).

**Fig 4:**
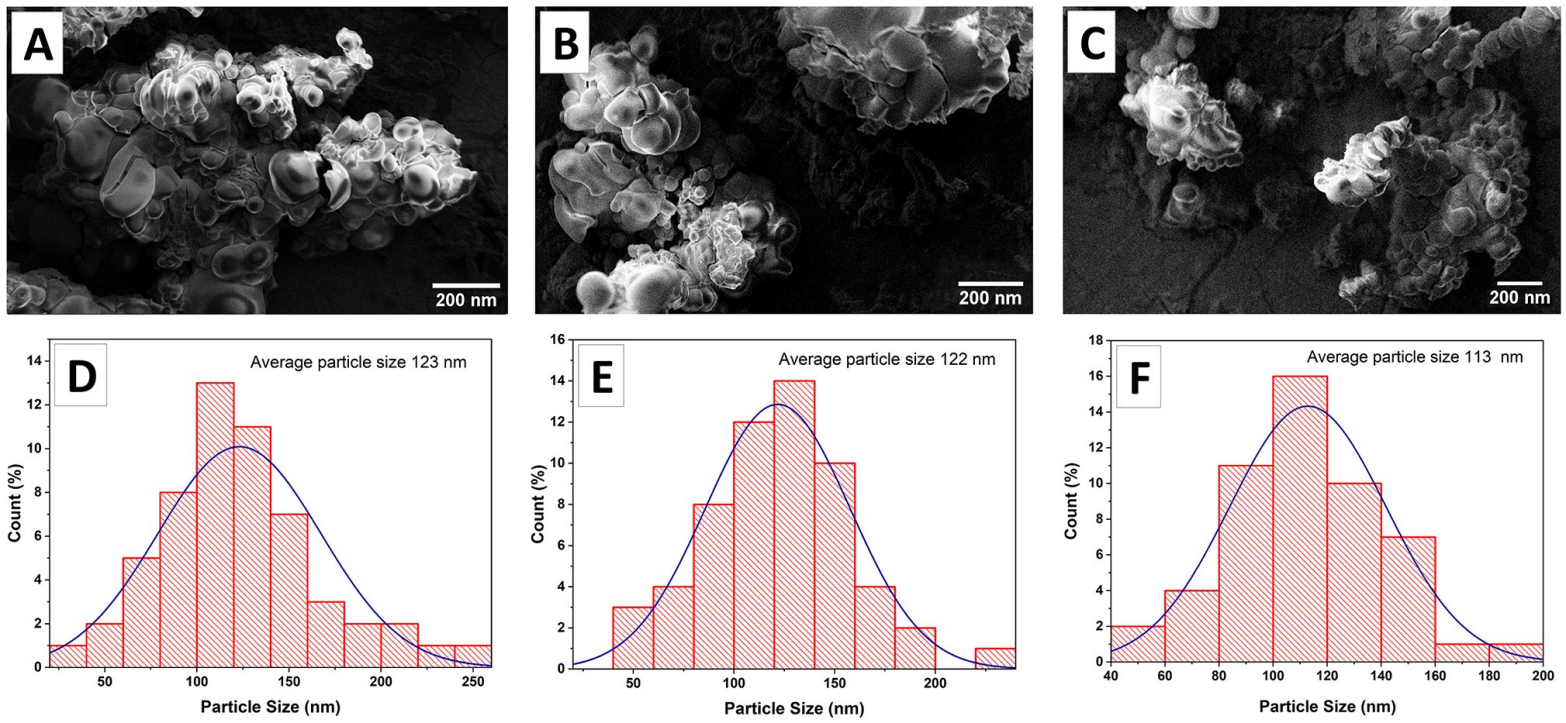
SEM image of MF59 _(OLE)_ (A), NLC-P _(OLE)_ (B) and NLC-L _(OLE)_ (C); Particles size distribution diagram of OLE-loaded nanoparticles MF59 _(OLE)_ (D), NLC-P _(OLE)_ (E), NLC-L _(OLE)_ (F).

The size and morphological characteristics of blank nanoparticles (Fig 3) and OLE-loaded nanoparticles (Fig 4) were evaluated by scanning electron microscopy (SEM). Electron microscope images were analyzed using OriginPro software (2018.SR1 v9.5.1).

As shown by SEM micrographs, the nanoparticles displayed round shapes, approximately uniform distribution of sizes, and smooth and almost homogeneous surfaces. Diameters estimated using scale bars were uniformly less than 150 nm for all nanocarriers, corroborating the results derived from DLS analysis.

#### Encapsulation efficiency

In this study, the concentration of OLE was determined based on the amount of oleuropein, which is the most abundant and effective polyphenol in OLE. The standard curve for OLE was generated at 234 nm (λmax of oleuropein). To measure the encapsulation efficiency, OLE-loaded nanoparticles were analyzed by two methods: centrifugation and membrane dialysis. The concentration of free OLE was then determined using a UV-visible spectrophotometer and the standard curve. The results showed encapsulation efficiency of OLE in MF59, NLC-P and NLC-L nanoparticles was 96.5, 95.8 and 93.7% respectively. These findings indicate MF59 exhibits a higher encapsulation efficiency for OLE compared to the NLC formulations.

#### In vitro drug release

**Fig 5:**
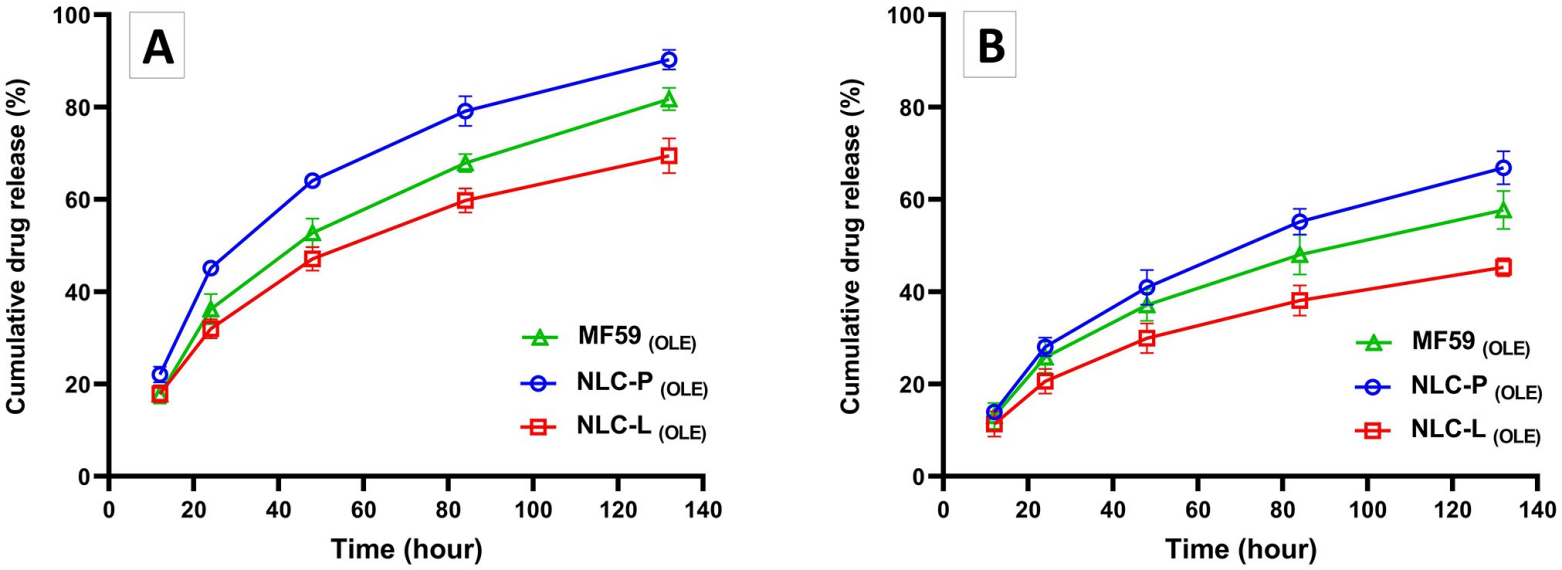
In vitro OLE release rate from MF59 _(OLE)_, NLC-P _(OLE)_ and NLC-L _(OLE)_ at pH 5.4 (A) and 7.4 (B). The findings indicate the mean (±SD) of three independent experiments.

To measure the OLE release from MF59, NLC-P, and NLC-L, OLE-loaded nanoparticles were analyzed at the specified time points using the dialysis membrane method at pH 5.4 and 7.4. The concentration of free OLE was determined through standard curve using UV-visible spectrophotometer at 234 nm. Fig 5 depicts the percentage of OLE release from the nanoparticles at designated time points in two graphs. The results demonstrated sustained release kinetics for OLE from MF59, NLC-P, and NLC-L nanoparticles; After 48 h, release was 53%, 64% and 47% at pH 5.4 and 37%, 41% and 30% at pH 7.4, respectively. These findings indicated that the release rate of OLE from NLC-P was greater than from MF59 and NLC-L nanoparticles under both pH conditions. Therefore, release was higher under acidic conditions mimicking the tumor microenvironment.

#### NDV infectious dose assay

The infectivity dose of the LaSota strain of NDV in HeLa cells was determined using the TCID50 assay. The viral titer was expressed as TCID50/mL and calculated using the Spearman & Kärber method (Hierholzer and Killington, 1996). The MOI value of the virus was calculated based on the TCID50 titer and was used as a measure to indicate the primary infection dose. Table 3 presents the results of TCID50/mL titer and corresponding plaque forming units PFU/mL) 0.69 × TCID50) for HeLa and HDF cell lines for 48 h post-infection. Considering the presence of approximately 40,000 HeLa cells per well and a PFU/mL titer of 157,000 at 48 h, an MOI of 0.4 was calculated for the LaSota strain of NDV.

**Table 3:** TCID50/mL and PFU/mL for HeLa and HDF cell lines in 48 h.

Cell line	HeLa		HDF	
Virus titration	TCID50/mL	PFU/mL	TCID50/mL	PFU/mL
48 h	**2.28 × 10** ^**5**^	**1.57 × 10** ^**5**^	**8.48 × 10** ^**2**^	**5.85 × 10** ^**2**^

#### Cytotoxicity analysis

**Fig 6:**
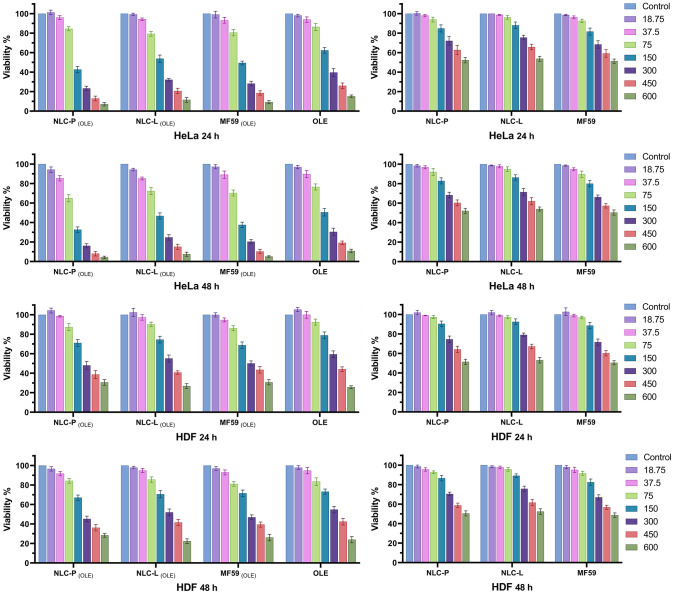
Effect of the 24 and 48 h treatments on cell viability in HeLa and HDF cells (OLE concentrations are in µM). The findings are shown as mean ± SD.

The MTT assay method was used to evaluate the cytotoxicity of empty nanoparticles, free OLE and its carrier nanoparticles, as well as the cytotoxicity of the LaSota strain of NDV, and also to investigate the combined effect of OLE-loaded nanoparticles and NDV in HeLa and HDF cells. Incremental concentrations ranging from 18.75 to 600 μM of OLE (equivalent to 10 to 324 μg/mL based on oleuropein concentration) and corresponding amounts of empty nanoparticles were tested. Cell viability relative to untreated controls was calculated as percentage for all groups. Treatments with at least three replicates were performed for different OLE concentrations in both cell lines at 24 and 48 h. Fig 6 shows the cell viability results from the treatments in HeLa and HDF cells. MTT results demonstrated the highest OLE cytotoxicity at 48 h, so this time point was used to investigate the effect of combined treatment.

The IC50 values were determined using GraphPad Prism v9.5.1 Software following the cell viability assay. Table 4 presents the findings, which demonstrate that NLC-P _(OLE)_ had the highest cytotoxicity followed by MF59 _(OLE)_, NLC-L _(OLE)_, and then free OLE at 48 h post-treatment.

**Table 4:** The IC50 value results of treatments of free OLE and its carrier nanoparticles in HeLa and HDF at 24 and 48 h.

Treatment groups		24 h		48 h	
Nano-carrier Type	Abbreviation Name	IC50 for HeLa cells (μM)	IC50 for HDF cells (μM)	IC50 for HeLa cells (μM)	IC50 for HDF cells (μM)
OLE-loaded NLC (Precirol)	NLC-P _(OLE)_	149.0	307.4	104.3	272.9
OLE-loaded NLC (Lecithin)	NLC-L _(OLE)_	177.2	331.8	137.2	301.1
OLE-loaded MF59	MF59 _(OLE)_	163.3	319.1	120.9	286.2
Free OLE	OLE	220.5	360.9	161.6	317.0
Blank NLC (Precirol)	NLC-P	656.8	628.5	638.5	605.1
Blank NLC (Lecithin)	NLC-L	685.0	658.1	667.2	637.3
Blank MF59	MF59	618.4	593.8	595.7	569.5

**Fig 7:**
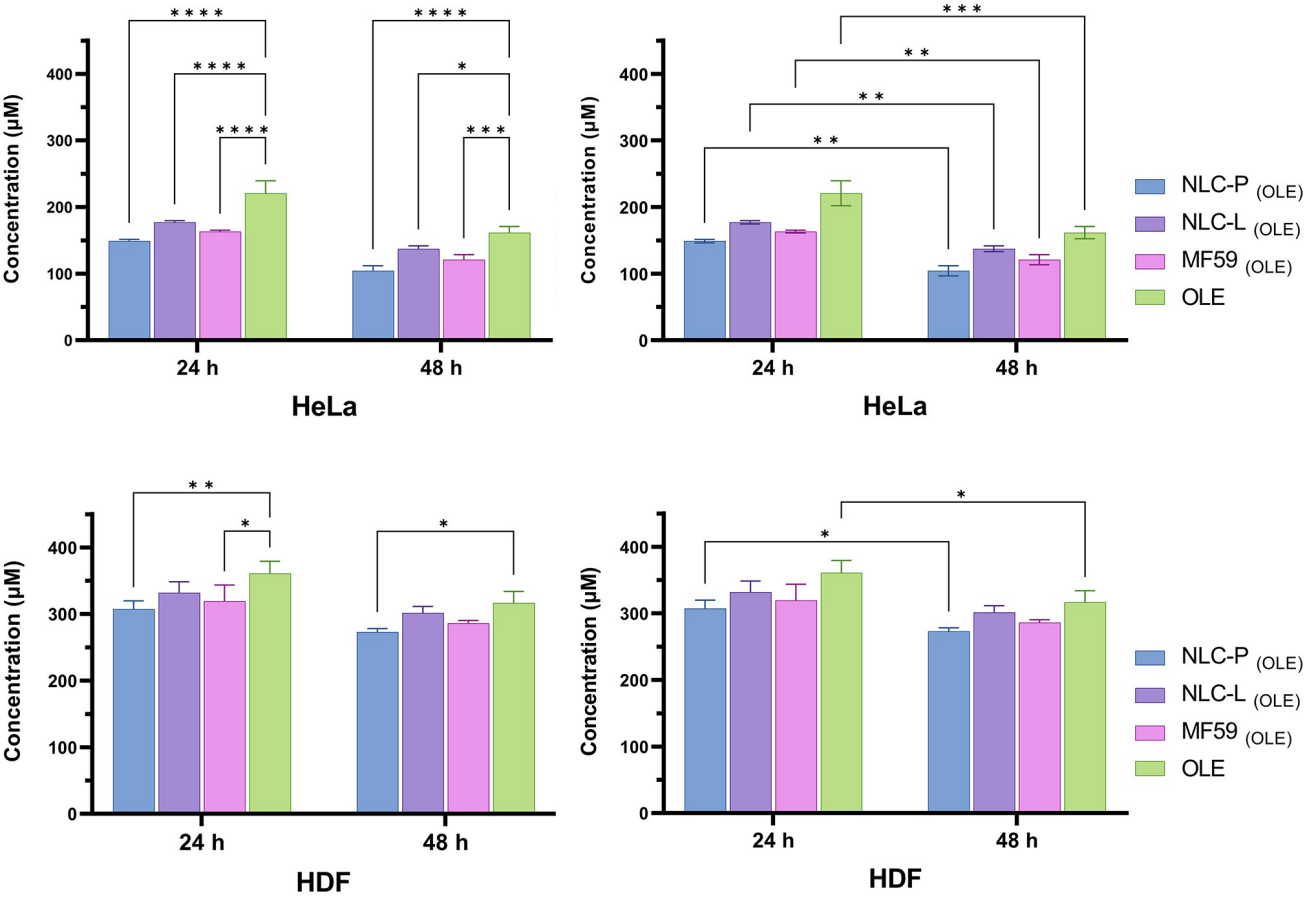
Comparison chart of groups treated with free OLE and its carrier nanoparticles in HeLa and HDF for 24 and 48 h. The findings indicate the mean (±SD) of three independent experiments.

Fig 7 shows a statistical comparison of the treatment groups in terms of IC50 values at 24 and 48 h post-treatment.

**Fig 8:**
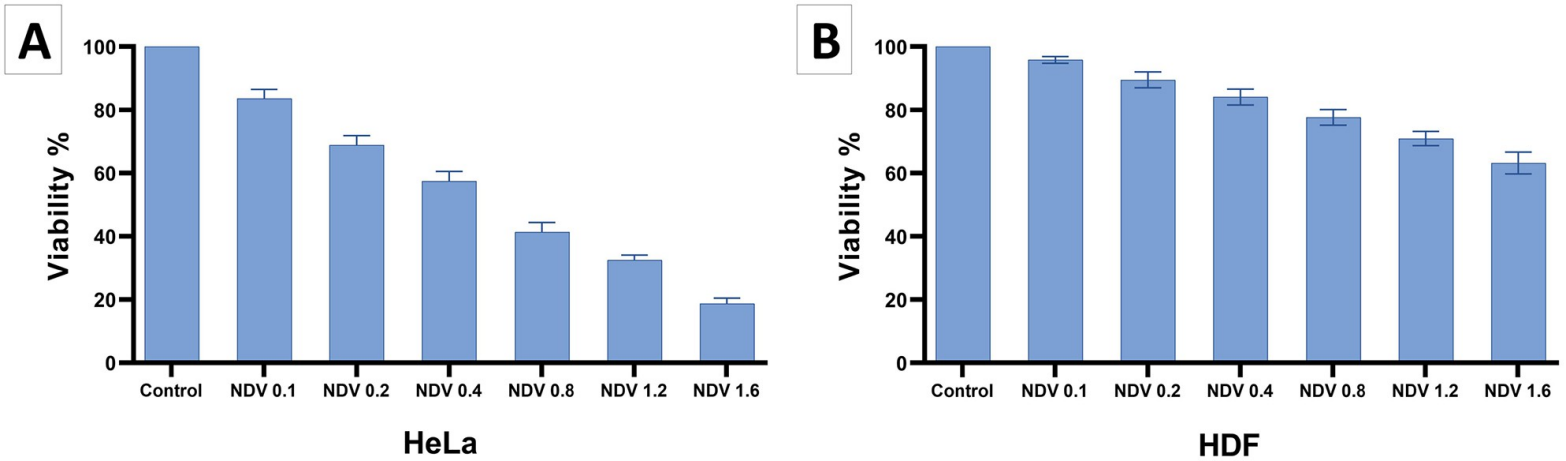
Diagrams of cytotoxicity assay of NDV (LaSota strain) at different MOI (0.1, 0.2, 0.4, 0.8, 1.2, 1.6) in HeLa (A) and HDF (B) cell lines for 48 h. The findings indicate the mean (±SD) of three independent experiments.

To evaluate the cytotoxicity of the virus, HeLa and HDF cell lines were infected with the LaSota strain of NDV at MOI of 0.1, 0.2, 0.4, 0.8, 1.2, and 1.6 for 48 h. Then IC50 value was calculated using GraphPad Prism v9.5.1 Software. Fig 8 shows the findings, indicating an IC50 value of 0.51 for the LaSota strain in HeLa cells and 3.33 in HDF cells at 48 h post-infection. Based on the lower IC50 value for cancer cells, an MOI of 0.4 was selected for NDV infection in the combined treatment study with OLE-loaded nanoparticles.

**Fig 9:**
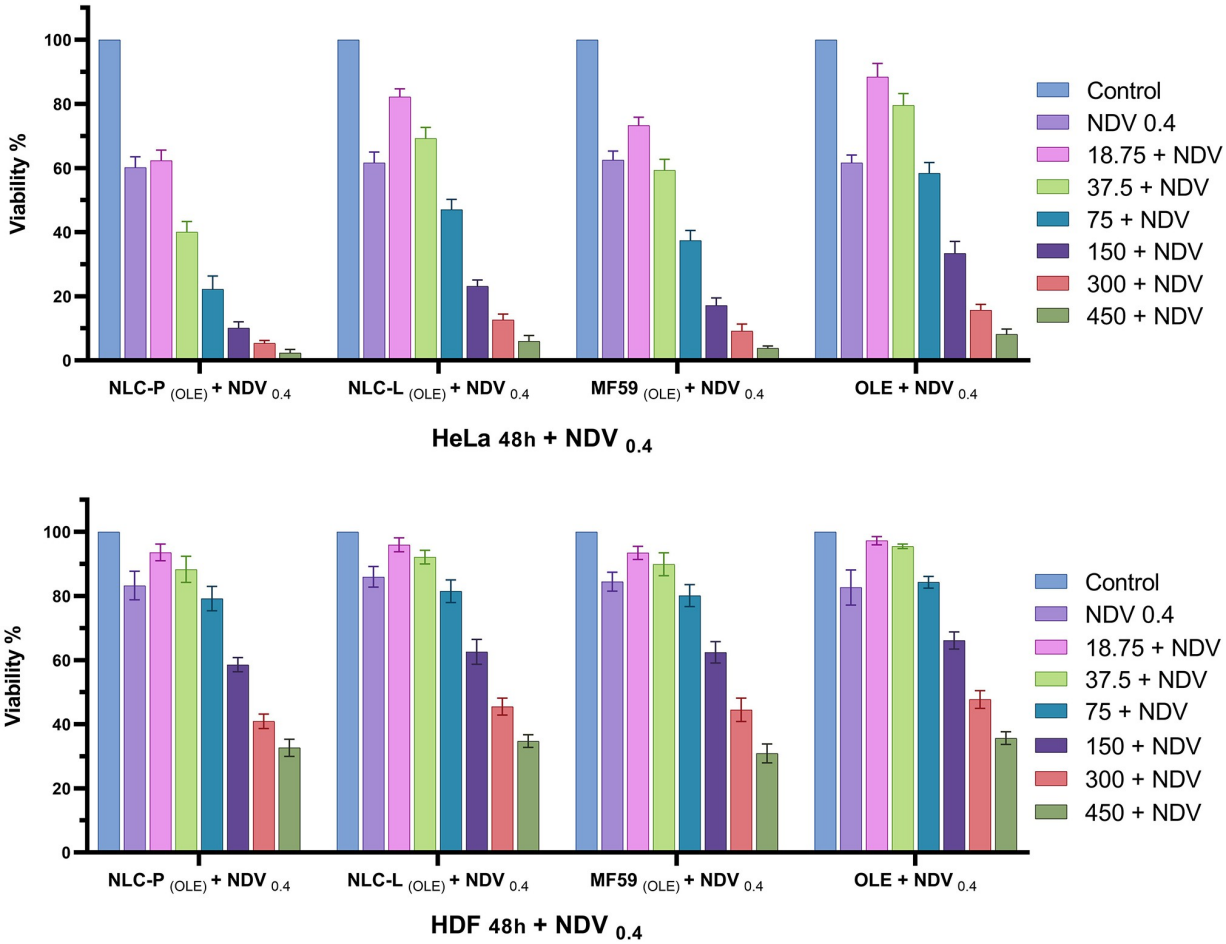
Cell viability outcomes of combination treatments in HeLa and HDF for 48 h. OLE concentrations are in µM and NDV at MOI = 0.4. The findings indicate the mean (±SD) of three independent experiments.

Finally, to investigate the cytotoxicity of the combined treatment, HeLa and HDF cell lines were treated with free OLE and OLE-loaded nanoparticles at concentrations of 18.75, 37.5, 75, 150, 300, and 450 μM along with infection by the LaSota strain of NDV at an MOI of 0.4 for 48 h. Fig 9 presents the cell viability results of these combination treatments in HeLa and HDF cells.

The IC50 values for the combination treatments were determined by nonlinear regression analysis of the cell viability data obtained from the MTT assay, using GraphPad Prism v9.5.1 Software. Table 5 presents the results, which showed that infection with NDV at an MOI of 0.4 in combination with NLC-P _(OLE)_ produced the greatest cytotoxic effect, indicated by the lowest IC50 value. This was followed by combinations with MF59 _(OLE)_, NLC-L _(OLE)_, and free OLE, respectively. These findings demonstrated that co-treatment with NLC-P _(OLE)_ and NDV at the selected dose achieved the highest synergistic cytotoxicity compared to other combinations tested in this study.

**Table 5:** The IC50 value results of combined treatments in HeLa and HDF at 48 h.

Treatments 48 h		NDV at MOI 0.4	
Nano-carrier Type	Abbreviation Name	IC50 for HeLa cells (μM)	IC50 for HDF cells (μM)
OLE-loaded NLC (Precirol) along with LaSota strain of NDV	NLC-P _(OLE)_ + NDV	27.7	222.0
OLE-loaded NLC (Lecithin) along with LaSota strain of NDV	NLC-L _(OLE)_ + NDV	66.0	254.5
OLE-loaded MF59 along with LaSota strain of NDV	MF59 _(OLE)_ + NDV	47.3	237.2
Free OLE along with LaSota strain of NDV	OLE + NDV	93.1	275.9

**Fig 10:**
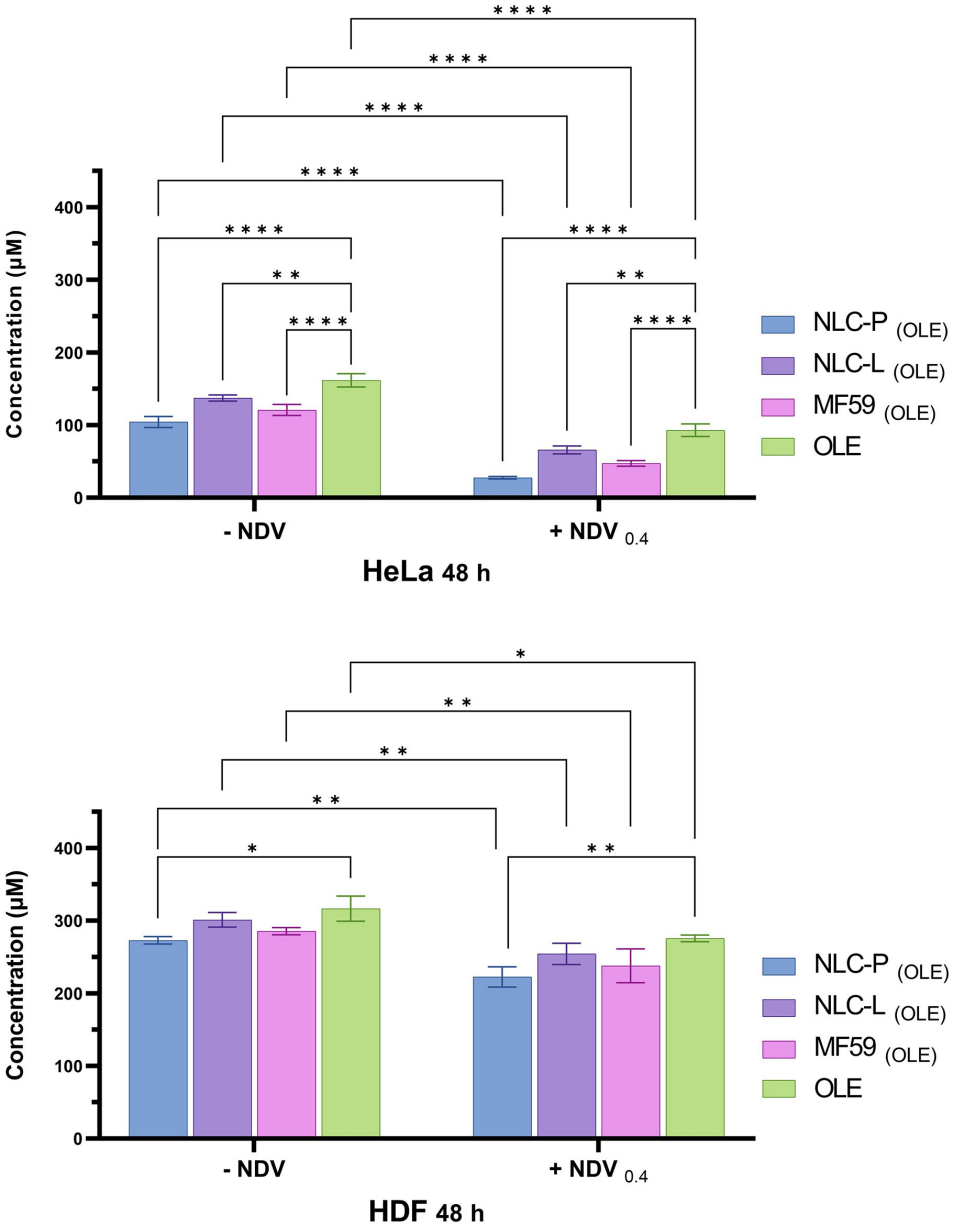
Comparison chart of groups with combination treatment in HeLa and HDF at 48 h. The findings indicate the mean (±SD) of three independent experiments.

A statistical comparison of the treatment groups in terms of their IC50 values from the combination therapy in HeLa and HDF cell lines at 48 h post-treatment is presented in Fig 10. The figure provides a visual assessment of the significant differences between IC50 values, which are an inverse measure of cytotoxic potency. This allows comparison of the relative synergistic cytotoxicity produced by each combination treatment against the two cell lines.

#### Apoptosis assay

**Fig 11:**
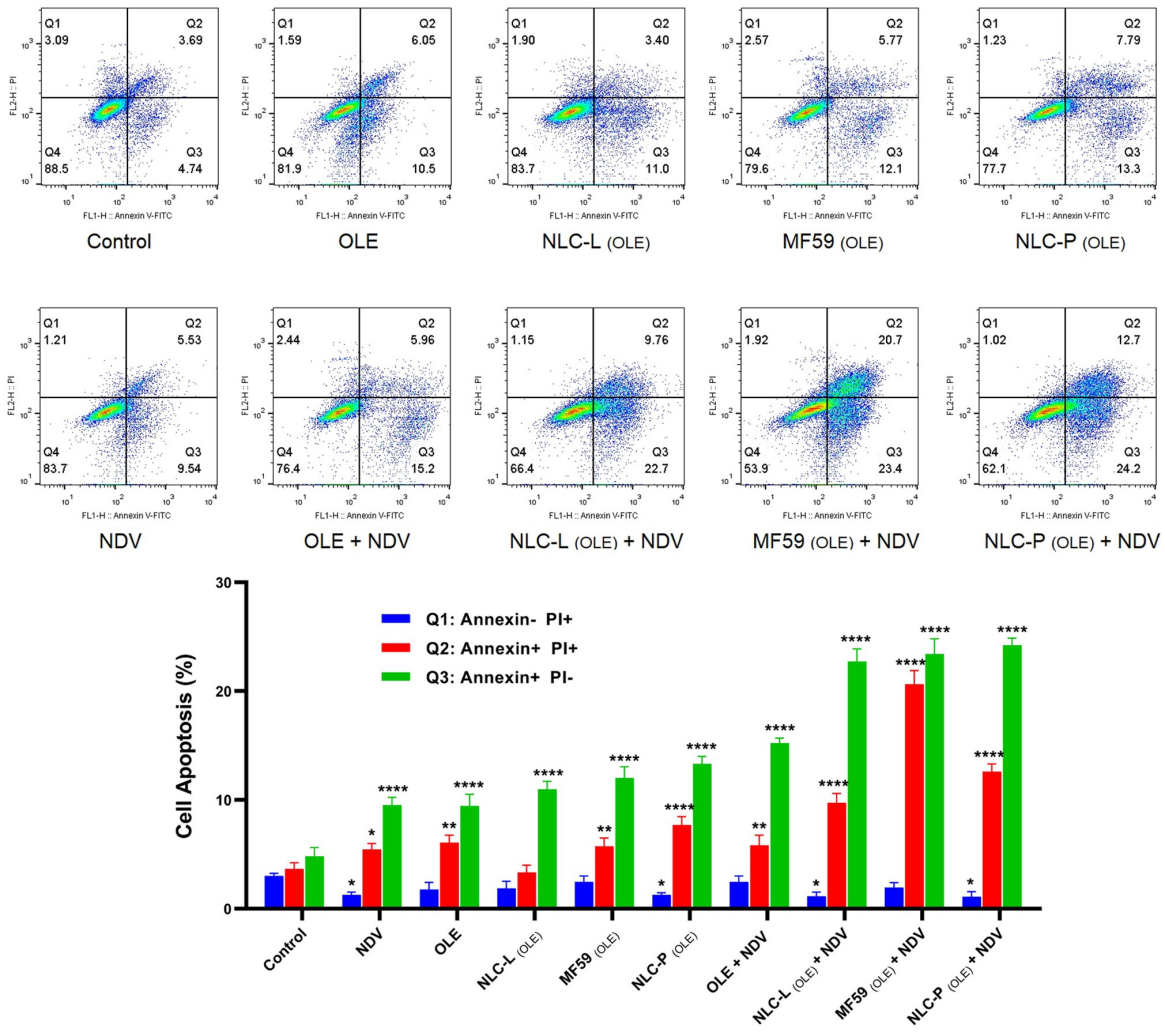
The results of apoptosis assay in the combined treatment of NDV at MOI 0.4 along with OLE and its carrier nanoparticles (100 μM) in HeLa cells at 48 h. Significance compared to control is shown as * (**:<0.05, ***:<0.001 and ****:<0.0001). The findings indicate the mean (±SD) of three independent experiments.

Induction of apoptosis in HeLa cells treated with different groups was assessed using Annexin V-FITC/PI staining analyzed by flow cytometer (BD FACSCalibur, USA). HeLa cells were infected with NDV and treated with free OLE and its carrier nanoparticles, separately and in combination for 48 h. Untreated cells served as controls. The data were analyzed using FlowJo software (v10.0.7) from three independent experimental replicates. Fig 11 illustrates the apoptotic outcomes of the indicated treatments. The highest percentage of early apoptosis was observed for the combination of NDV and NLC-P _(OLE)_, followed by combinations with MF59 _(OLE)_, NLC-L _(OLE)_, and free OLE. This suggests the NDV and NLC-P _(OLE)_ combination most effectively induced apoptotic cell death in HeLa cells.

#### Cell cycle assay

**Fig 12:**
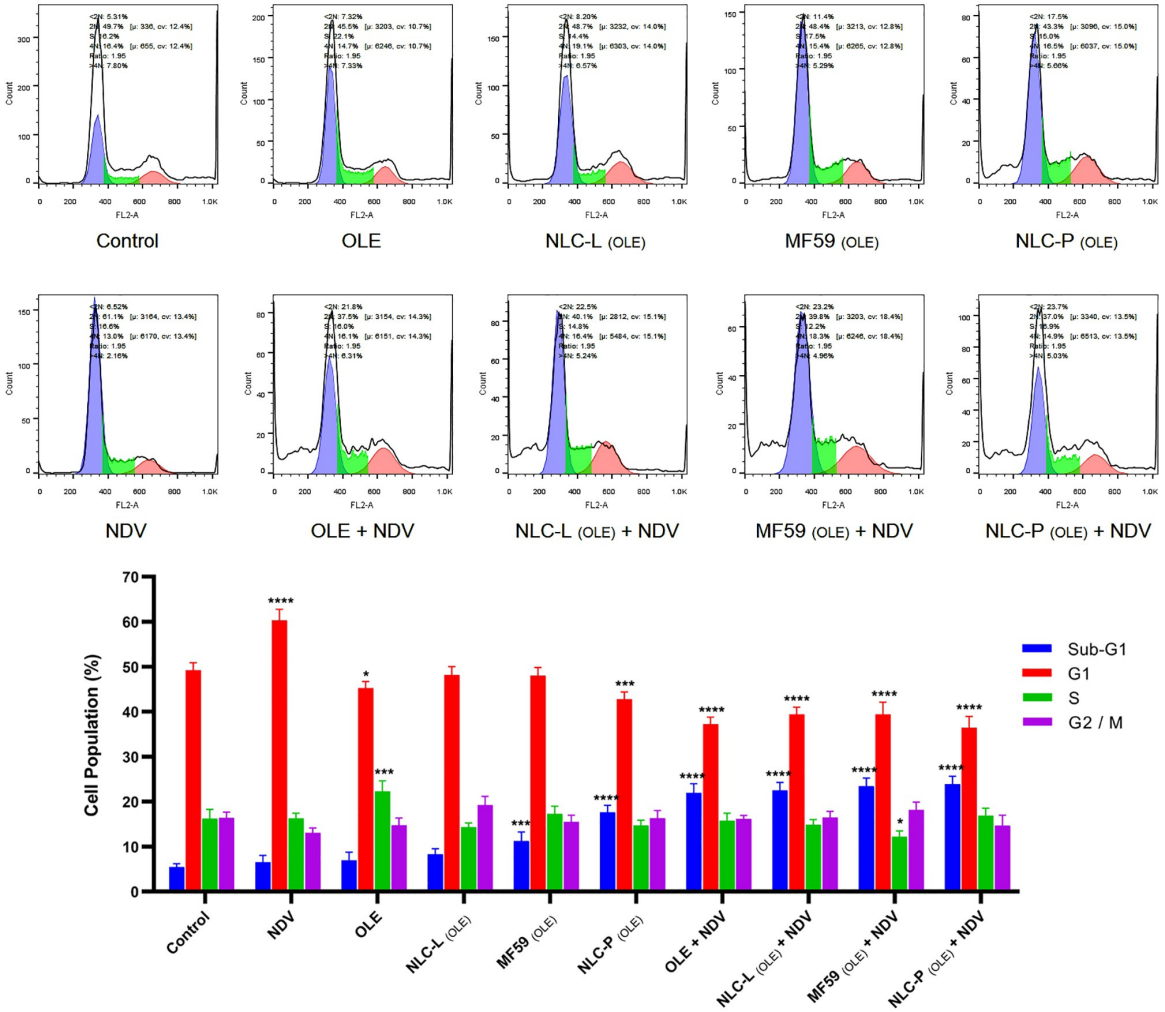
The results of cell cycle assay in the combined treatment of NDV at MOI 0.4 along with OLE and its carrier nanoparticles (100 μM) in HeLa cells at 48 h. Significance compared to control is shown as * (**:<0.05, ***:<0.001 and ****:<0.0001). The findings indicate the mean (±SD) of three independent experiments.

The dispensation of cell cycle phases in treated HeLa cells was evaluated using PI staining analyzed by flow cytometer (BD FACSCalibur, USA). As in the previous step, HeLa cells were infected with NDV and were treated with free OLE and its carrier nanoparticles, separately and in combination for 48 h. Untreated cells served as controls. Data from three independent experimental replicates were analyzed using FlowJo software (v10.0.7). Fig 12 shows the diagram and the cell cycle outcomes of the indicated treatments.

This experiment allowed evaluation of the relative effectiveness of different treatment groups in inducing cell cycle arrest in HeLa cells. The findings indicated that cell cycle arrest in treated HeLa cells occurred predominantly in the sub-G1 phase for all treatment groups. The accumulation of cells in the sub-G1 phase of the cell cycle is suggestive of cell death induced by apoptosis. This finding corroborates the previous observations of apoptosis as determined by Annexin V-FITC/PI staining.

## 4. Discussion

Cervical cancer is the fourth most frequently diagnosed cancer in women globally, and the second most prevalent cancer in developing nations, following breast cancer [1,2]. Since the 1970, human papillomavirus (HPV) has been established as the primary causative factor for cervical cancer development, while additional contributing factors such as environmental, biological, and hormonal influences are also involved [3,4]. The critical HPV oncoproteins, particularly E6 which degrades the tumor suppressor p53, and E7 which binds to the retinoblastoma protein (Rb), disrupt the cell cycle and proliferative processes of infected host cells. This eventually causes the cells to evade apoptosis and induce tumorigenesis [5]. The high mortality rate of this cancer in women, coupled with limitations of current treatment options, underscores the urgent need for safer and more efficacious therapeutic strategies against this disease. In recent years, due to the shortcomings of conventional cancer treatment modalities, the induction of apoptosis has emerged as a promising therapeutic approach to hinder the cancer cell proliferation and develop more potent anticancer drugs [6].

Several investigations have demonstrated that plant extracts rich in polyphenol compounds, including olive leaf extract (*Olea europaea*), exhibit anticancer activity and apoptosis induction in numerous cancer cell lines, such as leukemia cells [10], melanoma [29], breast cancer, and ovarian cancers [30], prostate cancer, and colorectal cancer [12], neuroblastoma [11]. The principal polyphenols present in olive leaves and fruits include oleuropein, hydroxytyrosol, tyrosol, oleosin, and oleocanthal, among which oleuropein represents the most abundant and bioactive constituent. Oleuropein displays potent antioxidant activity in normal cells. Additionally, several studies indicate that total polyphenolic extracts confer superior efficacy over individual phenolic components [9,10]. These polyphenols exert anticancer effects predominantly by modulating oxidative stress, cellular signaling transduction, and gene expression related to proliferation and apoptosis. Therapeutic benefits of olive leaf extract in cancer cells include: reducing mitochondrial membrane potential and function; increasing mitochondrial reactive oxygen species (ROS), which mediate pro-oxidant effects on cancer cells and represent a major anti-cancer mechanism causing cell cycle arrest, apoptosis induction, and cytotoxicity in cancer cells; upregulating pro-apoptotic proteins by cleaving poly (ADP-ribose) polymerase (PARP) and activating caspase 9 cascade leading to cancer cell apoptosis. Glucose transporters (GLUT) which are overexpressed on the surface of cancer cells, aid oleuropein binding and transport into cancer cells. Oleuropein and its derivatives hinder pro-survival Akt pathway signaling, which ultimately increases the level of pro-apoptotic proteins and decreases the levels of anti-apoptotic proteins [7,8,11,12].

However, the instability of OLE polyphenols in environmental and biological conditions and their relatively hydrophobic nature pose important challenges for their permeability and transport through cell membranes. Poor solubility, low bioavailability, and instability limit the therapeutic application of olive leaf extract. Nanoparticle encapsulation offers an approach to ameliorate these issues. MF59 is a nanoemulsion adjuvant that has been shown to enhance the immune response to vaccine antigens effectively. Previous studies have demonstrated the effectiveness of MF59 in improving encapsulation efficiency and controlled release of chemotherapeutic drugs. Due to its nanoscale particle size and ability to efficiently encapsulate and deliver bioactive compounds (such as OLE polyphenols), MF59 is an attractive candidate for developing drug delivery systems for cancer therapy [31]. Also, nanostructured lipid carriers (NLCs) are promising nanoparticles for encapsulated drug delivery to cancer cells, with advantages such as high stability, biocompatibility, non-toxicity, possibility Scale-up, high drug loading, sustained release, and enhanced local drug deposition [16]. The components utilized in NLC and MF59 formulations are biodegradable, non-stimulatory to the human immune system, and possess intrinsic anti-inflammatory effects [32]. Squalene is a natural component of humans and animals (in adipose tissue, skin, and muscles) and degrades rapidly in the environment [33]. Precirol ATO 5 is composed of glyceryl palmitostearate and shown to degrade >60% within 28 days in OECD biodegradability tests, confirming biodegradability [34]. Lecithin is a natural food emulsifier and breaks down to phosphatidylcholine, which is eliminated by the kidneys [35]. Polysorbates like Tween 80 undergo primary biodegradation >60% within 28 days according to OECD guidelines, classifying them as readily biodegradable [36]. Sorbitan esters such as Span 85 are inherently biodegradable, with degradation >60% achieved in standardized biodegradability tests [37].

In the present study, OLE was encapsulated inside the mentioned nanocarriers with the aim of enhancing stability, bioactivity, protecting against degradation and facilitating targeted delivery. OLE was encapsulated in MF59 and two NLC formulations (NLC-P and NLC-L) varying in composition. Physicochemical analyses were conducted to determine encapsulation efficiency, particle size, polydispersity index (PDI), zeta potential, and stability of the different formulations. In vitro release kinetics were evaluated to identify the carrier with the most sustained release profile at physiological (pH 7.4) and acidic (pH 5.5) pH mimicking tumor environments. Cytotoxicity against cancerous (HeLa) and normal (HDF) cell lines was assessed to select the carrier with the highest selective toxicity. Physicochemical characterization demonstrated the efficient performance of nanocarriers in terms of size, zeta potential, PDI, encapsulation efficiency, and release profile. NLC-P and MF59 exhibited superior performance compared to NLC-L, also all nanoparticles displayed sizes <150 nm and negative zeta potentials. PDI values were consistently below 0.4 for all nanoparticles, indicating homogeneous dispersion. The incorporation of span 85 and tween 80 surfactants is likely enhanced physical stability by preventing emulsion accumulation. Encapsulation efficiency surpassed 94% for all nanocarriers, with MF59 showing the highest efficiency. Release kinetics favored acidic pH conditions mimicking the tumor environments. Cytotoxicity assays conducted on HeLa and HDF cell lines showed significantly higher effects against cancer cells at 24 and 48 h (P<0.05). Among OLE-loaded nanocarriers, cytotoxicity decreased in the order of NLC-P > MF59 > NLC-L, correlating with the extent of OLE released from these nanocarriers. This trend was also supported by the differential cytotoxic responses induced by empty nanocarriers, albeit to a much lesser degree, with MF59 exhibiting the highest effect, followed by NLC-P and NLC-L, respectively.

Previous studies have investigated OLE encapsulation, including evaluation of the apoptotic effects of OLE-microcapsules against breast cancer and lung carcinoma [38], oleuropein encapsulation in NLC and its antioxidant effects in lung epithelial cells [16], and evaluation of the physicochemical properties of nanocarriers containing OLE [39]. These findings corroborate our results.

Oncolytic virotherapy shows promise as a cancer treatment approach. Natural or engineered oncolytic viruses have been utilized as a novel therapy by selectively replicating in and lysing cancer cells. These viruses have been in clinical use as oncolytic agents for over 30 years [18]. Principal oncolytic viruses identified include adenovirus, reovirus, herpes simplex virus, lentivirus, parvovirus, vaccinia virus, vesicular stomatitis virus, measles virus, and Newcastle disease virus (NDV). Deficiencies in cancer cell antiviral activity render them susceptible to viral infection, permitting viral survival. Tumor cells overexpress oncolytic virus receptors like sialic acid and EGFR, critical for viral transmission, replication, and initiation of endocytic signaling pathways. Oncolytic viruses induce localized inflammation through tumor microenvironment destruction while releasing viral progeny and tumor antigens to elicit systemic anti-tumor immunity. They can induce apoptosis in cancer cells with minimal side effects on normal cells [19]. NDV is an avian paramyxovirus that naturally displays tumor-selectivity and oncolysis. It possesses a single-stranded RNA genome encoding six genes. NDV oncolytic mechanisms involve induction of apoptosis through activation of various caspase-dependent apoptotic pathways (extrinsic and intrinsic) and expression of specific genes, stimulation of anti-cancer immune responses, and upregulation of p53 and ROS, which induce DNA damage and apoptosis [20,21]. Various studies have demonstrated NDV can directly penetrate cancer cells via hemagglutinin-neuraminidase (HN) and fusion (F) proteins without harming normal cells, leading to cancer cell lysis [22,23]. In this study, we employed the lentogenic LaSota strain of NDV as the oncolytic virus. LaSota cytotoxicity was evaluated in HeLa and HDF cell lines following MOI calculation. Based on 48 h MTT assay results, this virus exhibited significantly higher cytotoxicity against HeLa cancer cells than HDF normal cells (P<0.05), indicating its high tumor selectivity and potent anti-tumor activity. NDV oncolytic effects have been successfully investigated against different cancer types including breast [40,41], cervical [23], colorectal, and lung cancers [42], corroborating our findings.

As cancer is a heterogeneous, systemic disease, combination therapies provide an effective treatment strategy. Oncolytic virotherapy has demonstrated promising safety and efficacy, particularly when combined with standard anticancer interventions. Studies have shown successful synergistic effects for NDV combined with temozolomide-loaded nanoparticles against glioblastoma [43], investigation of NDV combined with vanadyl sulfate [44]. Combined NDV and rhubarb extract also enhanced oncolytic activity in cancer cells [45]. Combined NDV with rituximab and doxorubicin demonstrated synergistic cytotoxic effects against hematological malignancies [46]. Olive polyphenol combination therapies have also achieved substantial success against diverse cancers by augmenting drug effectiveness, improving tolerability, and reducing toxicities. Olive polyphenols synergized with cisplatin, tamoxifen, doxorubicin, mitomycin C, and paclitaxel [47].

In order to develop a safer and more effective cervical cancer treatment approach, we evaluated the combined impact of NDV LaSota strain and OLE-encapsulated lipid nanocarriers on HeLa cells in vitro. MOI of 0.4 reflected the LaSota IC50 range. Based on 48 h MTT assays, combination therapy cytotoxicity was significantly higher in HeLa versus HDF cells (P<0.05). Among OLE-encapsulated formulations in this combination treatment, NLC-P _(OLE)_, MF59 _(OLE)_, NLC-L _(OLE)_, and free OLE conferred descending cytotoxicity, respectively. As shown in Tables 4 and 5, in 48 h treatment, the IC50 values of OLE and nanocarriers containing it in Hela cancer cells were significantly reduced compared to their combination with NDV (for NLC-P _(OLE)_ from 104.3 to 27.7 μM, for MF59 _(OLE)_ from 120.9 to 47.3 μM, for NLC-L _(OLE)_ from 137.2 to 66.0 μM and for free OLE from 161.6 to 93.1 μM), with no difference observed in HDF cells. Comparisons of HeLa versus HDF cells were highly significant (p<0.05). In this combined treatment, the Annexin-V/PI apoptosis assay validated the MTT results, and cell cycle analysis demonstrated cell cycle arrest at G0/G1. A superior synergistic impact was observed against cancer cells versus normal cells. Comparing the ability of MF59 versus NLCs to encapsulate olive leaf extract, control release kinetics, and induce cytotoxicity provided important insight into the optimal nanocarrier system for further evaluation in combination with oncolytic virus therapy. This systematic approach allowed direct comparison of two leading lipid nanoparticle technologies to identify the formulation most suitable for co-delivery with NDV against cervical cancer cells.

## Conclusion

This study provides compelling evidence that co-delivery of NDV and OLE-loaded lipid nanoparticle formulations results in potentiated anticancer activity against cervical cancer cells. Physicochemical characterization confirmed successful OLE encapsulation within lipid nanocarriers, protecting its structural integrity and facilitating sustained release under physiological conditions. In vitro evaluations demonstrated significantly higher cytotoxicity and apoptotic effects of the combinatorial treatment, particularly with NLC-P _(OLE)_ and NDV, against HeLa cancer cells versus HDF normal cells. Flow cytometric analysis showed maximal cell cycle arrest in sub-G1 phase in cancer cells exposed to combination therapy. Overall, the observed synergistic anti-cervical cancer effects achieved through co-delivery of NDV and lipid nanoparticle-mediated OLE establish this approach as a promising alternative to conventional monotherapies warranting further exploration. Optimization of formulation variables and *in vivo* evaluation will help advance this combinatorial nanomedicine strategy toward clinical translation for effective cervical cancer management.

## Supporting information

S1 File SEM images and summary of OriginPro software results. (DOCX)

S2 File Data of encapsulation efficiency and OLE release. (XLSX)

S3 File The cell viability results of OLE treatments. (XLSX)

S4 File Cytotoxicity assay results of NDV at different MOIs. (XLSX)

S5 File The cell viability results of NDV combined with OLE treatments. (XLSX)

S6 File The results of apoptosis assay & cell cycle assay. (XLSX)

## Abbreviations

CPE: Cytopathic Effect
DLS: Dynamic light scattering
DMEM: Dulbecco’smodified Eagle medium
DMSO: Dimethyl sulfoxide
EE: Encapsulation Efficiency
FBS: Fetal bovine serum
FITC: Fluorescein isothiocyanate
FT-IR: Fourier transform infrared spectroscopy
HDF: Human Dermal Fibroblasts
HeLa: Human Cervical Carcinoma
HN: Hemagglutinin-neuraminidase glycoproteins
IC50: Inhibitory Concentration
MOI: Multiplicity of infection
NDV: Newcastle disease virus
NLCs: Nanostructured lipid carriers
OLE: Olive Leaf Extract
PBS: Phosphate-buffered saline
PDI: Polydispersity index
PI: Propidium iodide
Rb: Retinoblastoma protein
SEM: Scanning Electron Microscope
TCID50: Tissue culture infectious dose
