# Supplementary material for: Synergistic cytotoxicity of olive leaf extract-loaded lipid nanocarriers combined with Newcastle disease virus against cervical cancer cells
Source: PLoS One. 2024 Aug 14;19(8):e0308599. doi: 10.1371/journal.pone.0308599 (PMC11324187; doi:10.1371/journal.pone.0308599)

| N total | Mean | Standard Deviation | Sum | Minimum | Median | Maximum |
| --- | --- | --- | --- | --- | --- | --- |
| 50 | 112.2706 | 38.68977 | 5613.53 | 31.419 | 113.407 | 203.951 |

**Results MF59**


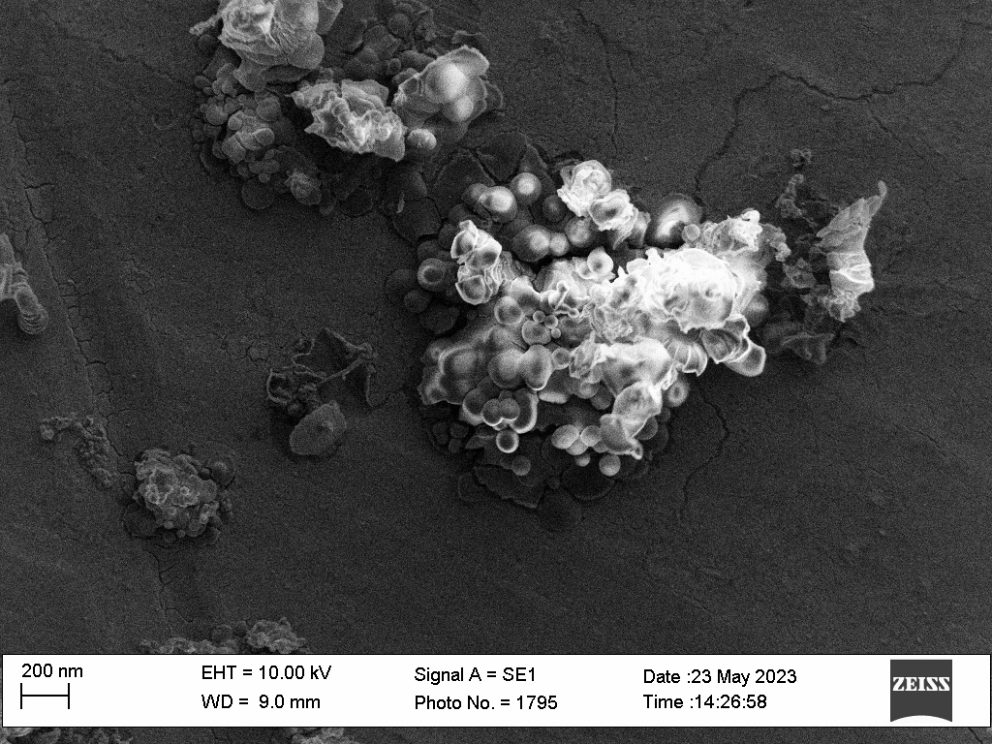


| N total | Mean | Standard Deviation | Sum | Minimum | Median | Maximum |
| --- | --- | --- | --- | --- | --- | --- |
| 48 | 109.0758 | 35.21098 | 5235.637 | 51.423 | 106.758 | 187.869 |

**Results NLC-P**

**
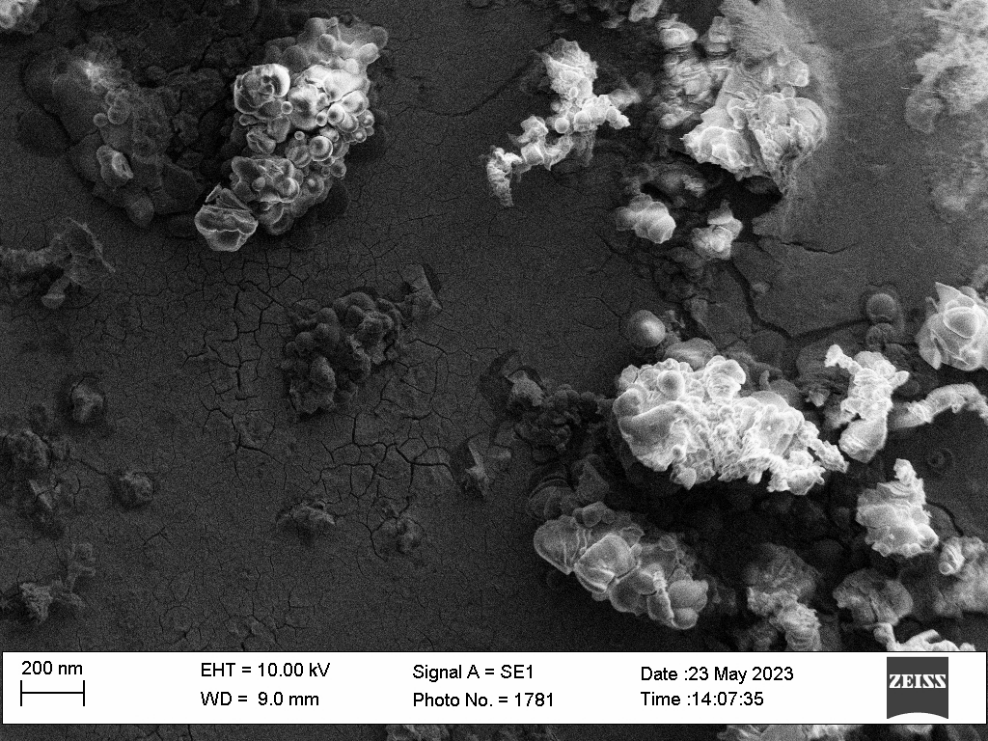
**

**Results NLC-L**

| N total | Mean | Standard Deviation | Sum | Minimum | Median | Maximum |
| --- | --- | --- | --- | --- | --- | --- |
| 48 | 104.882 | 32.6032 | 5034.334 | 39.16 | 101.8825 | 190.708 |

**
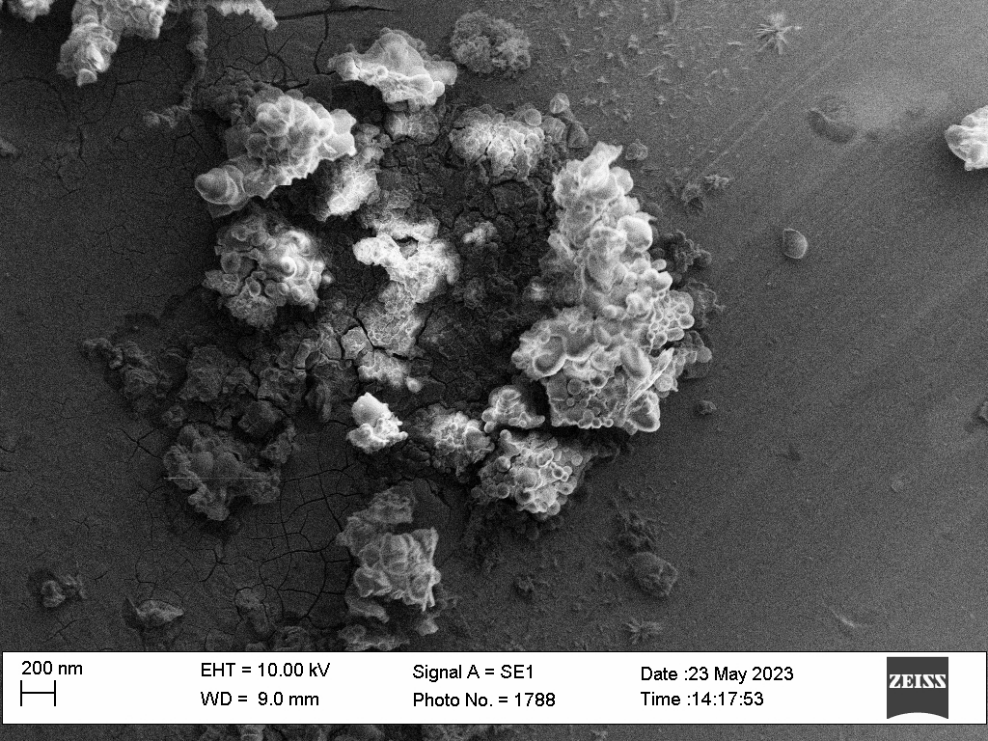
**

**Results MF59 _(OLE)_**

| N total | Mean | Standard Deviation | Sum | Minimum | Median | Maximum |
| --- | --- | --- | --- | --- | --- | --- |
| 56 | 123.079 | 44.27687 | 6892.425 | 33.273 | 117.4895 | 240.456 |

**
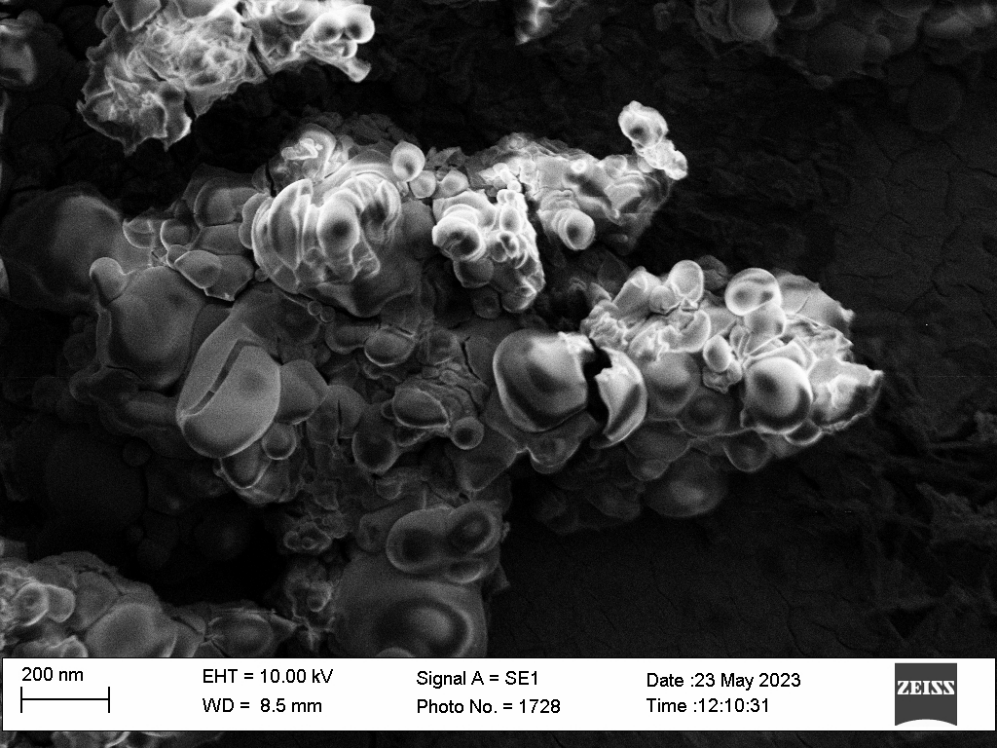
**

**Results NLC-P _(OLE)_**

| N total | Mean | Standard Deviation | Sum | Minimum | Median | Maximum |
| --- | --- | --- | --- | --- | --- | --- |
| 58 | 121.7024 | 35.97592 | 7058.741 | 41.501 | 121.389 | 223.706 |


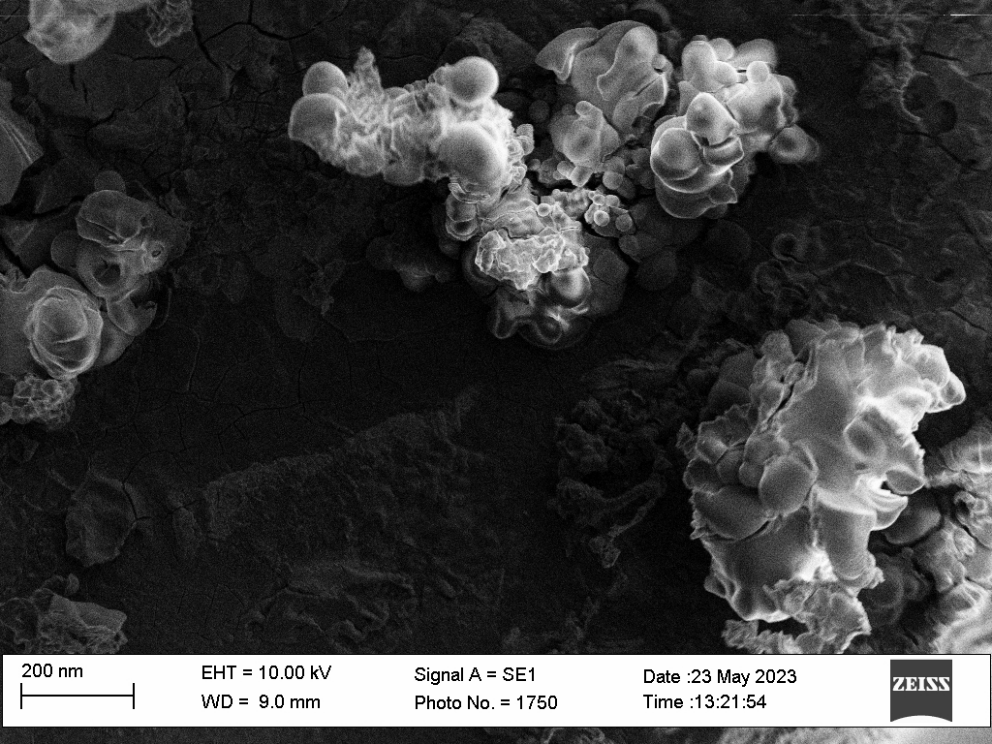


**Results** **NLC-L _(OLE)_**

| N total | Mean | Standard Deviation | Sum | Minimum | Median | Maximum |
| --- | --- | --- | --- | --- | --- | --- |
| 52 | 112.864 | 28.95175 | 5868.929 | 47.507 | 112.2295 | 185.924 |


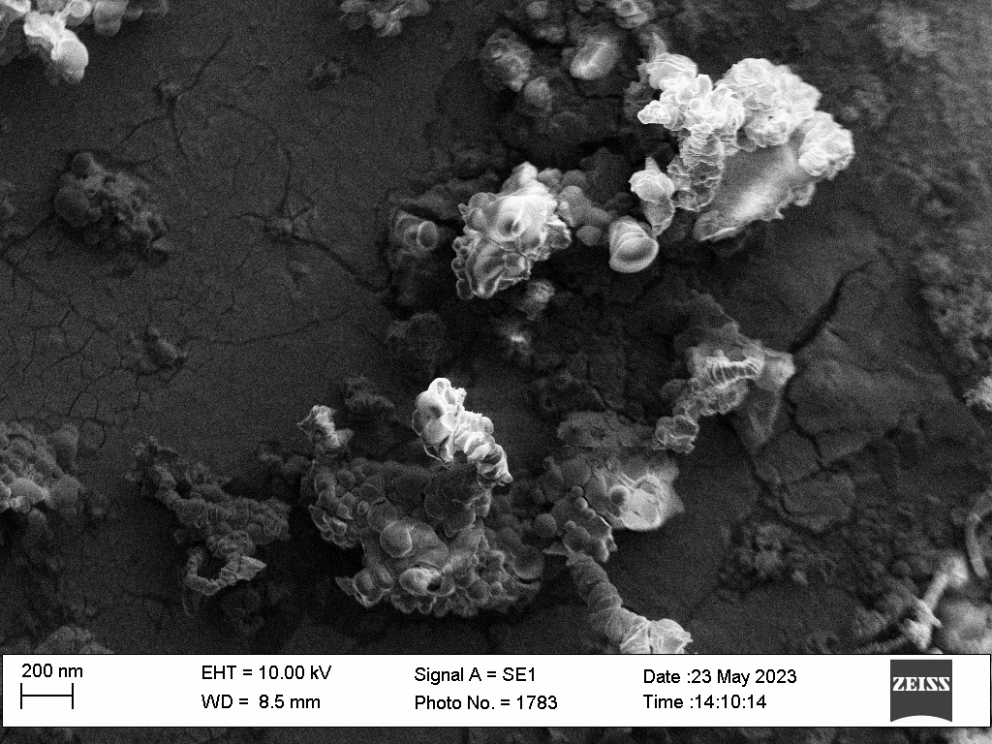

Supplement: S1 File — (DOCX) [file pone.0308599.s001.docx]
